# Risk Factors of Malnutrition among In-School Children and Adolescents in Developing Countries: A Scoping Review

**DOI:** 10.3390/children11040476

**Published:** 2024-04-15

**Authors:** Mustapha Amoadu, Susanna Aba Abraham, Abdul Karim Adams, William Akoto-Buabeng, Paul Obeng, John Elvis Hagan

**Affiliations:** 1 Department of Health, Physical Education and Recreation, University of Cape Coast, Cape Coast PMB TF0494, Ghana; mustapha.amoadu001@stu.ucc.edu.gh (M.A.); bdulkarimsaako2@gmail.com (A.K.A.); paul.obeng@stu.ucc.edu.gh (P.O.); 2 Department of Public Health, School of Nursing and Midwifery, College of Health and Allied Sciences, University of Cape Coast, Cape Coast PMB TF0494, Ghana; sabraham@ucc.edu.gh; 3 Department of Education and Psychology, University of Cape Coast, Cape Coast PMB TF0494, Ghana; wakotobuabeng@gmail.com; 4 Neurocognition and Action-Biomechanics-Research Group, Faculty of Psychology and Sports Science, Bielefeld University, Postfach 10 01 31, 33501 Bielefeld, Germany

**Keywords:** developing countries, education, health, in-school children, malnutrition, risk factors

## Abstract

Malnutrition among in-school children is a complex issue influenced by socio-economic, environmental, and health-related factors, posing significant challenges to their well-being and educational trajectories in developing countries. This review synthesized evidence on the multifaceted aspects of child malnutrition within the educational setting in developing countries. This review followed the six steps outlined by Arksey and O’Malley’s framework. Four main databases (PubMed, CENTRAL, JSTOR, and Scopus) were searched. Additional searches were conducted in WHO Library, ProQuest, HINARI, Google Scholar, and Google. Reference lists of eligible papers were checked. This review found that low family income, varying family sizes, parental employment status, and educational levels significantly impact malnutrition among in-school children and adolescents. Environmental elements, including rural/urban residence, household sanitation, and living conditions, also influence malnutrition. In addition, nutrition knowledge, dietary habits, nutrient deficiencies, physical activity, and prevalent health conditions compound the risk of malnutrition. This study underscores the extensive health impact of malnutrition on general health, specific nutrient deficiencies, fetal/maternal health concerns, and overall morbidity. Also, malnutrition affects school performance and attendance, impacting cognitive abilities, and academic achievements. Addressing these challenges requires comprehensive policy actions aligned with Sustainable Development Goals, emphasizing poverty alleviation, health literacy, and gender equity.

## 1. Introduction 

Malnutrition among in-school children in developing countries stands as a pressing global health concern, exerting far-reaching consequences on the well-being and prospects of these vulnerable populations [1]. Despite global efforts, the landscape of child malnutrition continues to evolve, marked by disparities and persistent threats to children and adolescents in developing nations [1,2]. Malnutrition is an inadequate or imbalanced intake of essential nutrients and it consists of both undernutrition and overnutrition [3]. Malnutrition encompasses both undernutrition, characterized by an insufficient intake of essential nutrients leading to deficiencies, and overnutrition, marked by excessive consumption often resulting in obesity and related health issues [3]. It reflects a spectrum of dietary imbalances that can have profound impacts on health and wellbeing. Its challenges compromise health, educational attainment, and overall human development [4,5,6], acting as a formidable barrier to achieving Sustainable Development Goals (SDGs), particularly those related to health, education, and poverty alleviation [2,7].

Undernutrition, typified by an insufficient nutrient intake and limited access to nourishing foods, remains a critical concern in various regions [1]. This silent crisis manifests in forms like stunting, wasting, and micronutrient deficiencies [1,3], impacting children’s susceptibility to infections, physical and cognitive development, and elevating the risk of morbidity and mortality [8]. Stunting, a consequence of chronic undernutrition, not only hampers physical growth but also compromises cognitive function, educational achievement, and future economic productivity [9]. Overnutrition refers to a condition in which an individual consumes more calories than their body requires for energy expenditure and metabolic functions [10]. This often leads to weight gain and can result in various health issues such as obesity, cardiovascular diseases, diabetes, and metabolic disorders [7,10]. Thus, overnutrition among school-age children in developing nations, manifests as conditions such as overweight and obesity, stemming from the consumption of excess calories, unbalanced dietary patterns, and sedentary behaviors [10]. This dietary imbalance exacerbates health concerns and underscores the urgent need for comprehensive interventions addressing both dietary habits and lifestyle choices. Furthermore, malnutrition in overweight individuals underscores the complexity of nutritional status, as excess weight does not necessarily equate to an adequate intake of essential nutrients, often masking underlying deficiencies and posing additional health risks [2,7,10]. This dual burden of malnutrition, with coexisting undernourished and over-nourished individuals, further complicates the global health landscape [2]. Overnutrition-related non-communicable diseases have emerged as significant threats among children and adolescents, emphasizing the urgency of addressing this issue [11].

The consequences of malnutrition, affecting health, education, and economic opportunities, have a significant impact on in-school children in developing countries [1]. Undernourished children grapple with weakened immune systems, illnesses, and stunted growth [12], affecting not just health but also permeating into educational realms. Stunted growth and cognitive impairments may diminish learning capacity, leading to poor academic performances [8]. Undernourishment’s impact on concentration, cognitive abilities, and increased absenteeism due to illness affects educational outcomes [13]. Overnutrition-related health issues also disrupt school attendance and performance [13], thus perpetuating a vicious cycle of poverty as inadequate education leads to economic hardships [14].

Despite the profound implications of malnutrition among in-school children in developing countries, a comprehensive understanding of risk factors, dynamics, and consequences remains lacking. While extensive research exists in various contexts, a dedicated review focusing on challenges faced by in-school children in developing countries is imperative. Thus, this scoping review aims to map studies on the risk factors, health impact and educational outcomes of malnutrition among in-school children and adolescents in developing countries. By systematically synthesizing the existing literature, this review aims to shed light on the multifaceted aspects of child malnutrition within educational settings. This scoping review offers insights into the depth of available evidence, highlights areas requiring further investigation, informs policy development, guides interventions, and ultimately contributes to enhancing the understanding and management of malnutrition among in-school children in developing nations. Additionally, by delineating the existing gaps in research, this review serves as a foundation for future studies and systematic reviews, fostering more targeted and impactful research endeavors. 

## 2. Methods

### 2.1. Study Design, Data Source and Search Strategy

This scoping review was based on Arksey and O’Malley’s [15] guidelines: (1) identifying and stating the research questions; (2) identifying relevant studies; (3) selecting the studies; (4) collecting data; (5) data summary and synthesis of results; and (6) consultation. The following questions guided this review: (1) What are the risk factors of malnutrition among in-school children and adolescents in developing countries? (2) What is the health impact of malnutrition among in-school children and adolescents in developing countries? (3) What is the impact of malnutrition on educational outcomes of in-school children and adolescents in developing countries?

Four main databases (PubMed, Scopus, Central, and JSTOR) were searched for relevant studies. Medical Subject Heading (MeSH) terms were utilized for the search in PubMed and refined for the search in other databases. The search strategy conducted in PubMed is presented in Table 1. The MeSH terms and search strategy were modified for search conducted in other databases. The authors scrutinized the records obtained, and the Mendeley software version 1.19.8 was used to remove duplicates. The WHO Library, ProQuest, HINARI, Google Scholar, Google and institutional repositories of universities in Ghana were searched for additional records. Furthermore, reference lists of eligible records were checked for other relevant articles. The last search was conducted on 4 November 2023. 

In the first phase of the screening process, the Mendeley software was used to remove duplicate records. Furthermore, 12 graduate students were trained and supervised by MA to screen titles and abstracts for full-text records. This level of screening was guided by the eligibility criteria presented in Table 2. In the final phase of the screening process, full-text records identified were screened for inclusion against the eligibility criteria. This was carried out independently by two authors (PO and AKA). This process was supervised by MA. These processes were conducted to ensure objectivity in the screening process and to make sure all relevant records were screened. 

### 2.2. Data Extraction

The data extraction form was developed and piloted independently by PO and AKA to extract data from three included studies. The piloting was carried out to ensure reliability, reduce bias, identify discrepancies, and enhance validity in capturing essential information from the three studies. Details that were extracted during the data charting process include authors and year, study design, population, sample size, prevalence, risk factors and study recommendations. Details of extracted data are presented in Table S1. In addition, we consulted a review and subject experts to ensure the accuracy and depth of data for this scoping review. Finally, thematic analysis and synthesis were carried out, and the narrative results were presented and summarized in tables.

## 3. Results

### 3.1. Search Outcomes

**Figure 1:**
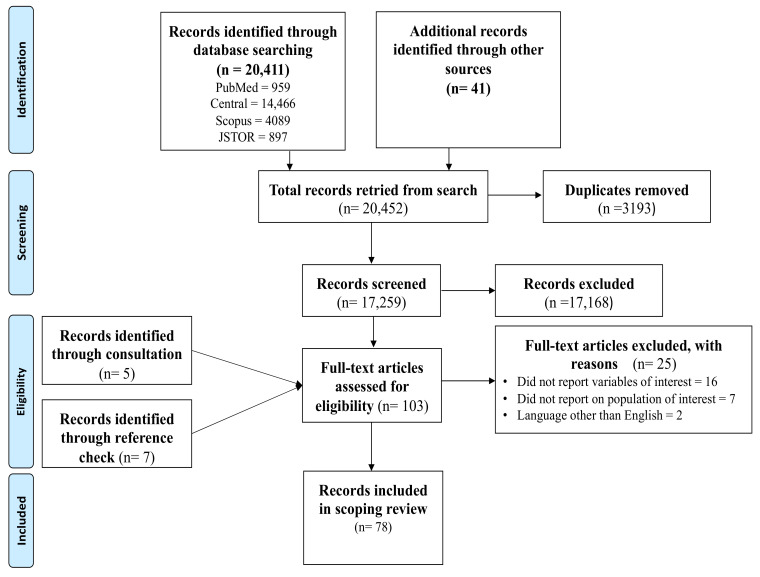
Prisma flow-chart of search results and screening process.

The initial search conducted across the four main databases yielded a total of 20,411 records. This number was complemented by an additional 41 records from other online sources. The Mendeley software was used to eliminate 3193 records. The screening of the title led to the exclusion of 17,168 records. Thus, 91 full-text eligible records were left for further screening. An additional five records were obtained through consultation with a librarian, and seven more records were discovered by scrutinizing the reference lists of the full-text eligible records. Finally, 103 full-text records underwent screening against the predetermined eligibility criteria. Ultimately, 78 full-text records met the inclusion criteria and were included in this scoping review. Refer to Figure 1 for detailed information on the screening process. 

### 3.2. Characteristics of Included Studies 

**Figure 2:**
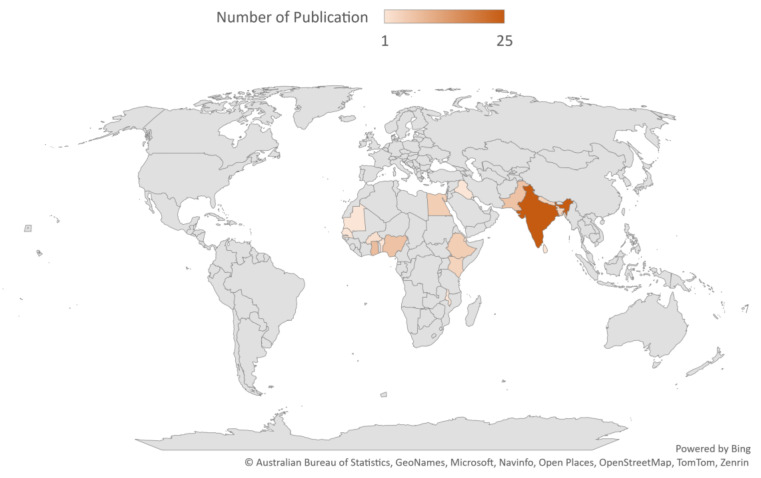
Country of publication and number of publications.

Most of the included studies were conducted in India (n = 25), followed by Ghana (n = 8) (see Figure 2 for details). The majority of the studies included in this review used cross-sectional survey design (n = 76). The rest were experimental (1) and case–control (1) studies. See the Supplementary Table (Table S1) for details on extracted data from included studies.

### 3.3. Risk Factors of Malnutrition among In-School Children in Developing Countries’ Demographics 

Various demographic factors influenced malnutrition among in-school children in developing countries. Low family income [16,17,18,19,20,21,22,23,24,25,26,27,28,29,30,31,32,33,34,35,36,37,38,39,40,41,42,43,44] and higher socio-economic status [28] were identified to contribute to malnutrition among in-school children and adolescents. Additionally, nuclear family [44] and large family size [16,21,24,30,32,33,36,37,38,39,41,45,46,47] contributed to the risk of malnutrition. Furthermore, unemployment [46], irregular work of the father [30], maternal unemployment [39], parental occupation as laborers [20,48] and being dependent on agricultural land [16] were found to be associated with malnutrition among in-school children and adolescents. Also, low educational status of parents [17,21,23,26,29,32,33,34,36,37,43,44,45,46,48,49,50,51,52,53] and parents with formal education [54] have been reported as risk factors for malnutrition among school going children and adolescents in developing countries. Factors such as the timing of introduction to complementary foods [16], the type of school attended [42], age (both younger [17,41,47] and older [55,56]), gender (both female [39,43] and male [38,47]), being from a male-headed household during adolescence [17], and maternal loss [46] were identified as contributing to the risk of malnutrition. 

#### 3.3.1. Nature of Environment and Sanitation 

The environment in which children lived contributed to the risk of malnutrition. For instance, the studies included reported that living in flood-prone areas [18], poor environments [26], and slum [36,57] were associated with higher malnutrition rates. Notwithstanding, living in either rural [28] or urban [28,44,58] environments or living in small houses [39] had a significant impact on malnutrition as well. Sanitation issues, such as unsafe drinking water [16,54] and poor personal hygiene [59] were also linked to malnutrition among in-school children and adolescents in developing countries.

#### 3.3.2. Knowledge of Practice 

Insufficient knowledge about nutrition [20,23,24,60,61], poor breastfeeding practices [16] and early cessation of breast feeding [52] were reported as risk factors for malnutrition. Also, a lack of awareness about nutritional needs was reported as a risk factor for malnutrition among in-school children and adolescents in developing countries [60].

#### 3.3.3. Dietary Habits 

In-school children and adolescents with poor eating habits [36] such as regular consumption of carbonated soft drinks [13,62], consumption of fast foods [13,62], low consumption of high-quality protein [20,30,41,56,63,64,65,66,67], high consumption of low-diversity diets [17,25,68], habit of eating in between meals [69] were more prone to malnutrition. Also, in-school children and adolescents who regularly consumed beverages between meals per day [54], had dinner as the heaviest meal of the day [69], consumed more than three meals in a day [69] and skipped breakfast [30,56,70] were more prone to malnutrition. However, in-school children and adolescents who consumed foods such as plus, legumes, and lentils [71] were reported to be at risk of malnutrition. The studies included reported that food insecurity [13,37,43,50,62], inadequate dietary intake [17,27,31,42,47,52,63,65,67,68], and poor dietary quality contributed to malnutrition risk [13,17,25,50,67,69].

#### 3.3.4. Nutrient Deficiency and Body Weight

Malnutrition risk was associated with various nutrient deficiencies, including vitamin A [41,65,66,72], calcium [65,66], zinc [41,66,72], iodine [72], and iron [66,72]. Additionally, micro-nutrient deficiency [52,73] and maternal malnutrition [52] also contributed to malnutrition risk [52]. Furthermore, in-school children and adolescents who are obese or overweight were found to have an increased risk of malnutrition [74].

#### 3.3.5. Activity Level 

A study reported that using vehicular transport to school, lack of participation in household activities, children who watched TV more than 3 h per day and children who had the habit of not playing outdoor games were at increased risk of malnutrition [69]. According to other studies, children who depended on family members for household activities [60] and in-school children and adolescents who did not do any physical activities [62] were at elevated risk of malnutrition. 

#### 3.3.6. Malaise 

Certain health issues, such as diarrhea [68], cold/coughs [68] and anorexia [52], contributed to malnutrition risk. The risk factors of malnutrition among in-school children in developing countries are presented in Table 3.

### 3.4. Health Impact of Malnutrition among In-School Children in Developing Countries

#### 3.4.1. General Health Status 

Malnutrition among in-school children in developing countries significantly impacts their general health status. David et al. [20] found associations between malnutrition and various health issues such as malaria, headaches, nose bleeding, abdominal pains, fainting, diarrhea, colds/coughs, vomiting, and fever. Mwaniki et al. [68] also found that colds/coughs, vomiting and fever were associated with malnutrition among in-school children and adolescents in developing countries. Additionally, eye-related complications, including conjunctival xerosis [35], Bitot’s spot [35], myopia [59], and pallor [59], highlight the systemic impact of malnutrition on children’s health. An association has also been reported between malnutrition and dental caries [59,63,76].

#### 3.4.2. Fetal/Maternal Health and Morbidity 

The repercussions of malnutrition extend to fetal and maternal health, emphasizing the intergenerational impact of nutritional deficiencies. Ahmed et al. [13] identified poor maternal health, preterm birth, and an increased risk of being Small for Gestational Age (SGA) as outcomes associated with malnutrition during childhood. Additionally, malnourished children exhibited compromised immunity [28], leading to lowered resistance to infection [52,61], recurring illnesses [13], upper respiratory tract infections [20,76], lower respiratory tract infections [76], skin infections [20,68], parasitic infections [40,76] and an increased risk of non-communicable diseases [21,69]. Furthermore, Danquah et al. [67] reported increased morbidity in children suffering from malnutrition with physiological and developmental delays [28], faltering growth [61,64] and long-term relative physical growth retardation [28].

#### 3.4.3. Gender Discrimination

Beyond the physiological impact, malnutrition is also influenced by social factors. Gender discrimination, as identified by Khanam and Haque [16], exacerbated the health disparities among in-school children. The vulnerability of certain gender groups to malnutrition adds a social dimension to the multifaceted nature of this health issue [16]. 

#### 3.4.4. Nutritional Deficiency and Specific Body Systems 

Nutritional deficiencies contributed significantly to the health impact of malnutrition. Studies have emphasized the association between malnutrition and deficiencies in vitamin A [18,19,22,35,40], vitamin B-complex [18], iron [18,22,40,66,73], and calcium [63]. These deficiencies manifest in various health issues, including impaired cognitive functions [28,50,52,64,70,72,74]. Other metabolic impacts identified included obesity [21,64,74], overweight [21,74] and risk of nutrition-related chronic diseases [33]. Table 4 below presents the health impact of malnutrition among in-school children in developing countries.

### 3.5. Impact of Malnutrition on School Performance and Attendance of In-School Children in Developing Countries 

#### 3.5.1. School Performance 

Malnutrition among in-school children in developing countries is intricately linked to diminished learning ability [18,61] and work efficiency [18]. A study highlighted the negative impact of malnutrition on cognitive functions such as poor memory [52]. Moreover, malnourished children were more likely to experience poor academic performance [27,32,42,52,53,67,70,77,78,79] and other academic challenges such as a lack of concentration [32] and poor memory [52], which further hindered the overall educational experience of malnourished children. Recognizing these specific academic difficulties is crucial for implementing targeted interventions that address the cognitive impact of malnutrition and improve overall school performance.

#### 3.5.2. School Attendance 

Malnutrition significantly contributes to high levels of school dropout [20,32,42,74] and low-class attendance [32,42,64,72,74,80] among in-school children in developing countries. The consequences of malnutrition extended to delayed school entry [27,32,42] and decreased graduation rates from primary and secondary school, as highlighted by Roba et al. [27]. Details are presented in Table 5.

## 4. Discussion

### 4.1. Summary of Findings

The risk factors contributing to malnutrition among in-school children in developing countries span across diverse domains. Demographic factors, including low family income, higher socioeconomic status, nuclear or large family sizes, parental unemployment or specific occupational statuses, and educational status of parents, significantly influence the prevalence of malnutrition. Environmental aspects such as the type of residence (rural/urban), household sanitation, and living conditions also play pivotal roles. Knowledge of nutrition, dietary habits, nutrient deficiencies, physical activity levels, and prevailing health conditions further compound the risk. The health impact of malnutrition encompasses a wide array of issues, from compromised general health status and specific nutrient deficiencies to fetal/maternal health and morbidity. Moreover, malnutrition exerts a substantial toll on school performance and attendance, affecting cognitive functions, academic achievements, and overall educational trajectories. 

### 4.2. Risk Factors of Malnutrition among In-School Children in Developing Countries

In developing countries, the complex web of risk factors contributing to malnutrition among in-school children is deeply rooted in socio-economic disparities, environmental challenges, inadequate healthcare access, and lifestyle dynamics [27,81]. These regions often grapple with widespread poverty, leading to limited economic resources within households [16,17]. Families facing financial constraints struggle to afford nutritious food, access healthcare services, and provide a conducive environment for their children’s well-being [44]. The cyclical nature of poverty perpetuates malnutrition as it restricts educational opportunities, perpetuates unemployment, and limits access to diverse, quality diets [20,61].

Environmental factors, such as living in flood-prone areas, inadequate sanitation, and lack of access to clean water, are prevalent in many developing regions [18,36]. These challenges significantly impact children’s health, making them more susceptible to diseases that further exacerbate malnutrition [1]. In such contexts, efforts to improve living conditions by providing clean water, sanitation facilities, and safe housing are crucial to combatting malnutrition [82,83,84]. Healthcare-related findings underscore systemic issues related to knowledge gaps, inadequate healthcare access, and cultural practices [52,60]. Insufficient knowledge about proper nutrition and suboptimal breastfeeding practices perpetuate malnutrition. In many cases, limited access to healthcare facilities and health education exacerbates these challenges, indicating a need for accessible and culturally sensitive healthcare interventions [85,86].

Dietary habits among in-school children in developing countries reflect both economic constraints and cultural preferences [17,20]. Food insecurity, dietary monotony, and an inadequate intake of essential nutrients are often rooted in limited access to diverse and nutritious food sources [1,6]. The lack of dietary diversity is not only a consequence of poverty but also influenced by cultural habits and food availability. Additionally, sedentary lifestyles and limited physical activity among children compound the issue of malnutrition [68,69]. This stems from various factors, including inadequate infrastructure for recreational activities, safety concerns in outdoor spaces, and a lack of awareness regarding the importance of physical exercise [10,69].

### 4.3. Health Impact of Malnutrition on In-School Children in Developing Countries

The health impact of malnutrition among in-school children in developing countries is multifaceted, influencing various aspects of their well-being [1]. One crucial aspect is the general health status of these children, where malnutrition has been linked to a plethora of health issues ranging from gastrointestinal problems like diarrhea and vomiting to respiratory ailments such as colds/coughs and even fever [2,7,13]. These health challenges not only compromise their immediate health but also contribute to the complexity of managing their nutritional status [68]. The prevalence of these health issues indicates the vulnerability of malnourished children to a spectrum of illnesses, which is exacerbated by the challenging socio-economic conditions prevalent in developing countries [5,39].

The impact of malnutrition transcends childhood, extending to fetal and maternal health. Studies have highlighted the intergenerational consequences of malnutrition, associating it with poor maternal health, preterm birth, and an increased risk of SGA infants [13,76]. Compromised immunity in malnourished children leads to increased susceptibility to infections, including both communicable and non-communicable diseases, emphasizing the long-term health risks associated with childhood malnutrition [21,61]. Additionally, malnutrition contributes to delayed physiological and developmental milestones, stunting growth and development [67], further highlighting the pervasive impact on both short- and long-term health [1,81].

Nutritional deficiencies play a pivotal role in shaping the health consequences of malnutrition. Deficiencies in vitamins A, B-complex, iron, and calcium are prevalent among malnourished children, leading to a myriad of health issues, including impaired cognitive functions and increased risk of obesity, overweight, and chronic diseases [18,22,66,74]. These deficiencies exacerbate the health challenges faced by in-school children in developing countries, often due to limited access to diverse and nutritious diets, further exacerbated by socio-economic constraints. Gender discrimination exacerbates the health disparities among these children, and Khanam and Haque [16] shed light on the social dimensions of malnutrition. This underscores the need for tailored interventions addressing not only nutritional deficiencies but also societal and cultural factors influencing access to adequate nutrition and healthcare among vulnerable groups [1,87,88,89].

### 4.4. Impact of Malnutrition on Educational Outcomes of In-School Children in Developing Countries

Malnutrition among in-school children in developing countries has profound implications for their academic performance and attendance, which are crucial factors shaping their educational journey [7]. Malnutrition significantly impacts cognitive abilities, leading to diminished learning capacity and work efficiency among affected children [18,61]. The ramifications extend to academic challenges, including poor memory, lack of concentration, and overall underachievement [32,52]. These difficulties impede effective learning experiences, hindering educational progress and perpetuating academic disadvantages for malnourished students [7]. Recognizing these specific academic hurdles is vital for tailoring interventions that specifically address the cognitive repercussions of malnutrition and enhance overall school performance [77,78,80].

The consequences of malnutrition extend beyond academic hurdles to profoundly impact school attendance. Studies reveal a strong association between malnutrition and heightened school dropout rates, low class attendance, and delayed school entry among in-school children in developing nations [77,78]. These repercussions further culminate in decreased graduation rates from primary and secondary education [27]. The significance of addressing nutritional needs becomes evident in the urgency to enhance attendance rates, mitigate dropout tendencies, and secure a more fruitful educational path for these children [78]. The unique findings regarding academic challenges and attendance issues stem from multifaceted factors prevalent in developing countries. Socio-economic disparities, inadequate access to proper nutrition, and healthcare constraints often persist, making it challenging for families to provide balanced nutrition for their children [1]. Additionally, insufficient government interventions and a lack of emphasis on nutritional needs contribute to perpetuating cycles of malnutrition and its educational ramifications [1].

### 4.5. Limitations

This review’s focus on the published literature might introduce publication bias, potentially overlooking valuable unpublished studies. Additionally, the restriction to English language articles might have excluded insightful research published in other languages, possibly limiting the inclusivity of findings. Furthermore, the eligibility criteria’s specificity and keyword restrictions might have excluded pertinent studies, impacting the review’s comprehensiveness. Another potential limitation is the predominance of cross-sectional studies, which could influence the depth of the synthesized evidence. Cross-sectional surveys may be affected by bias responses, and this might affect the generalization of the findings of this review. The focus of this review on in-school children may mean that the findings, conclusions, and recommendations drawn from this study cannot be generalized to the general population of children, including out-of-school and homeless children. Despite these limitations, this review’s strength lies in its comprehensive synthesis of diverse studies from various databases, offering valuable insights into the prevalence and risk factors of malnutrition among in-school children and adolescents in Ghana. Additionally, the rigorous screening and consultation processes employed enhance the validity and reliability of the findings, reinforcing the relevance of the conclusions and recommendations drawn from the included studies.

### 4.6. Implications for Policy and Practice

The multifaceted risk factors contributing to malnutrition among in-school children in developing countries demand comprehensive policy measures and practical interventions to address this pressing issue. These findings hold significant implications for policy formulation, interventions, and aligning strategies with the SDGs to alleviate the impact of malnutrition and improve children’s well-being and educational outcomes [1]. Policy interventions should aim to alleviate poverty by implementing income support programs, livelihood opportunities, and social safety nets. Promoting nutritional literacy among parents, communities, and schools can empower individuals to make informed dietary choices. Increasing access to affordable healthcare services, particularly in rural areas, is crucial to address health disparities and provide early nutritional interventions [35,58]. Efforts should focus on improving sanitation, ensuring access to clean water, and creating healthier living conditions to mitigate health risks associated with malnutrition [1]. Implementing subsidized or free meal programs in schools can ensure children have access to nutritious meals, alleviating the economic burden on families and improving dietary intake in developing economies [11,40,62].

Targeted interventions should address cultural and societal factors contributing to malnutrition, particularly gender-based disparities. Tailored initiatives can empower girls and vulnerable groups, reducing discrimination and ensuring equal access to education and nutrition [28,44]. Advocacy for policy integration across sectors, including health, education, and social welfare, is essential for a holistic approach to combat malnutrition. Collaborative efforts between governments, NGOs, and international organizations are crucial for funding, implementing, and monitoring the effectiveness of interventions aimed at reducing malnutrition among in-school children. Practical solutions involve community-based programs that educate caregivers about optimal nutrition, offer guidance on cultivating home gardens for diverse food sources, and provide access to fortified foods or supplements for vulnerable groups [90,91,92]. Strengthening the health infrastructure in remote areas and training healthcare workers to identify and address malnutrition early can significantly impact children’s health outcomes [1,93,94]. By aligning policies, fostering cross-sector partnerships, and implementing practical interventions, governments and organizations can significantly reduce the prevalence of malnutrition among in-school children in developing countries, contributing to improved health and educational outcomes, ultimately advancing progress towards the SDGs [1,7,93,94].

### 4.7. Recommendations for Future Studies

Future studies should aim to address the gaps identified in this review by exploring the longitudinal impact of malnutrition on in-school children in developing countries. Longitudinal studies could provide valuable insights into the trajectory and long-term effects of malnutrition on health, educational outcomes, and overall well-being. Additionally, there is a need for more interventional studies that assess the effectiveness of targeted interventions in mitigating malnutrition among in-school children. These interventions could range from community-based nutritional programs to policy-driven initiatives aiming to enhance access to quality nutrition and healthcare services. Furthermore, future reviews should consider broadening their scope beyond English-language publications to encompass research published in other languages, ensuring a more comprehensive understanding of the global landscape of malnutrition among in-school children. Additionally, employing a mix of study designs, including qualitative approaches, can offer a more nuanced understanding of the socio-cultural factors influencing malnutrition and its impact. Lastly, incorporating diverse populations within developing countries would contribute to a more inclusive understanding of the multifaceted challenges associated with malnutrition not just among in-school children but also out of -school children.

## 5. Conclusions

The pervasive issue of malnutrition among in-school children in developing countries encompasses a complex interplay of socio-economic, environmental, and health-related factors. These multifaceted risk factors, rooted in poverty, inadequate healthcare access, environmental challenges, and lifestyle dynamics, significantly impact the nutritional status and overall well-being of children. The health implications span from immediate gastrointestinal and respiratory ailments to long-term consequences affecting maternal health, intergenerational health, and cognitive development. Malnutrition’s profound impact extends beyond health, detrimentally affecting educational outcomes, including cognitive abilities, academic performance, and school attendance, perpetuating cycles of disadvantage among vulnerable populations. Addressing these challenges demands comprehensive policy measures aligned with the SDGs, advocating for poverty alleviation, nutritional literacy, improved healthcare access, sanitation, and gender equality. Tailored interventions, community-based programs, and collaborative efforts across sectors and international organizations are imperative to mitigate malnutrition’s detrimental effects on in-school children. While this review offers valuable insights, future studies should focus on longitudinal research, interventional studies, and broader inclusivity in populations and languages to deepen our understanding and enhance effective strategies to combat malnutrition among these vulnerable groups.

## Figures and Tables

**Table 1:** Planned search strategy in PubMed.

Item	Search Strategy
**#1 Search to identify malnutrition**	Malnutrition [Meshi Term] OR Undernutrition OR Overnutrition OR Undernourishment OR Overnourishment OR Nutritional Deficiency OR Nutrient Deficiency OR Protein-Energy Malnutrition OR PEM OR Micronutrient Deficiency OR Vitamin Deficiency OR Mineral Deficiency OR Macronutrient Deficiency OR Energy Deficiency OR Caloric Deficiency OR Dietary Insufficiency OR Inadequate Nutrition OR Poor Nutrition OR Inadequate Intake OR Inadequate Diet OR Dietary Deficiency OR Food Insufficiency OR Food Scarcity OR Food Shortage OR Starvation OR Hunger OR Famine OR Wasting OR Stunting OR Underweight OR Overweight OR Obesity OR Body Mass Index OR BMI OR Kwashiorkor OR Marasmus OR Growth Failure OR Growth Stunting OR Growth Retardation OR Iodine Deficiency OR Iron Deficiency OR Vitamin A Deficiency OR Zinc Deficiency OR Calcium Deficiency OR Anemia
**#2 Search to identify risk factors**	Risk factors [MeSH Term] OR Determinants OR Causes OR Contributors OR Influences OR Precursors OR Triggers OR Antecedents OR Exposures OR Vulnerabilities OR Predictors OR Indicators OR Correlates
**#3 Search to identify health outcomes**	Health Outcomes [MeSH Term] OR Health Status OR Health Conditions OR Health Results OR Health Effects OR Health Impact OR Health Consequences OR Health Indicators OR Immune System Diseases OR Immune System Abnormalities OR Immune System Disorders OR Immune System Diseases OR Growth Disorders OR Growth Retardation OR Growth Impairment OR Growth Failure OR Anemia OR Iron-Deficiency Anemia OR Iron Deficiency Anemias OR Osteoporosis OR Bone Diseases, Metabolic OR Bone Demineralization, Pathologic OR Bone Loss OR Night Blindness OR Vision Disorders OR Cardiovascular Diseases OR Heart Diseases OR Hypertension OR Mental Disorders OR Mental Health OR Depression OR Anxiety Disorders OR Cognitive Dysfunction OR Mortality OR Mortality Risk
**#4 Search to identify school outcomes**	Education Outcomes [MeSH Term] OR School Outcomes [MeSH Term] OR Drop-out OR Truancy OR Skipping class OR Violence OR Poor academic performance OR Inattentiveness OR Educational Failure OR Academic Failure OR School Disengagement OR School Problems OR Poor school performance OR Poor educational attainment
**#5 Search to identify in-school children**	School Children [MeSH Term] OR Child OR Childhood OR Infants OR Toddlers OR Adolescents OR Adolescence OR Junior High School Students OR Senior High School Students OR Elementary School Students OR Preschoolers OR Preteens OR Pre-adolescents OR Middle School Students OR High School Students OR Primary School Students OR Nursery School Children OR Kindergarteners OR Grade School Children OR Teenagers OR Teenage Years OR Puberty OR Pre-schoolers OR Early Childhood OR Middle Childhood OR Late Childhood OR Youth
**#6 Search to identify developing countries**	Developing countries [MeSH Term] Africa OR Asia OR south America OR Afghanistan OR Albania OR Algeria OR Angola OR Antigua and Barbuda OR Argentina OR Armenia OR Azerbaijan OR Bahamas OR Bahrain OR Bangladesh OR Barbados OR Belarus OR Belize OR Benin OR Bhutan OR Bolivia OR Bosnia and Herzegovina OR Botswana OR Brazil OR Brunei OR Bulgaria OR Burkina Faso OR Burundi OR Cambodia OR Cameroon OR Cape Verde OR Central African Republic OR Chad OR Chile OR China OR Colombia OR Comoros OR Congo OR Costa Rica OR Côte d’Ivoire OR Croatia OR Cuba OR Cyprus OR Czech Republic OR Djibouti OR Dominica OR Dominican Republic OR Ecuador OR Egypt OR El Salvador OR Equatorial Guinea OR Eritrea OR Eswatini OR Ethiopia OR Fiji OR Gabon OR Gambia OR Georgia OR Ghana OR Grenada OR Guatemala OR Guinea OR Guinea-Bissau OR Guyana OR Haiti OR Honduras OR Hungary OR India OR Indonesia OR Iran OR Iraq OR Jamaica OR Jordan OR Kazakhstan OR Kenya OR Kiribati OR Kosovo OR Kuwait OR Kyrgyzstan OR Laos OR Latvia OR Lebanon OR Lesotho OR Liberia OR Libya OR Lithuania OR Macedonia OR Madagascar OR Malawi OR Malaysia OR Maldives OR Mali OR Malta OR Marshall Islands OR Mauritania OR Mauritius OR Mexico OR Micronesia OR Moldova OR Mongolia OR Montenegro OR Morocco OR Mozambique OR Myanmar OR Namibia OR Nauru OR Nepal OR Nicaragua OR Niger OR Nigeria OR Oman OR Pakistan OR Palau OR Palestine OR Panama OR Papua New Guinea OR Paraguay OR Peru OR Philippines OR Poland OR Portugal OR Qatar OR Romania OR Russia OR Rwanda OR Saint Kitts and Nevis OR Saint Lucia OR Saint Vincent and the Grenadines OR Samoa OR Sao Tome and Principe OR Saudi Arabia OR Senegal OR Serbia OR Seychelles OR Sierra Leone OR Solomon Islands OR Somalia OR South Africa OR South Sudan OR Sri Lanka OR Sudan OR Suriname OR Syria OR Tajikistan OR Tanzania OR Thailand OR Timor-Leste OR Togo OR Tonga OR Trinidad and Tobago OR Tunisia OR Turkey OR Turkmenistan OR Tuvalu OR Uganda OR Ukraine OR United Arab Emirates OR Uruguay OR Uzbekistan OR Vanuatu OR Venezuela OR Vietnam OR Yemen OR Zambia OR Zimbabwe
**Overall search strategy**	#2 AND #1 AND #5 AND #6 Not animal * #3 AND #1 AND 5 AND #6 Not animal * #4 AND #1 AND 5 AND #6 Not animal *
**Filters activated**	Language: English language Date: From 1 January 2000

* is used to stress on “Not animal” during search to take out all studies that used animals as samples.

**Table 2:** Eligibility criteria.

Inclusion criteria	The paper should be: - a peer-reviewed article, thesis, or dissertation; - published in 2000 or later; - published in the English language; - conducted on school-going children and adolescents (12–19 years); - on risk factors and impact of malnutrition.
Exclusion criteria	The paper should be: - conducted on out-of-school children and adolescents; - conducted on students over 20 years of age; - conducted outside developing countries; - a study published online before the year 2000; - a report, review, abstract, minutes, commentary, letter to editors, preprint, literature review; - outside the variables of interest.

**Table 3:** Risk factors of malnutrition among in-school children in developing countries.

Main Theme	Subtheme	Authors
Socio-demographics	Low total family income	[16,17,18,19,20,21,22,23,24,25,26,27,28,29,30,31,32,33,34,35,36,37,38,39,41,42,43,44,52,75]
	Higher socio-economic status	[28]
	Large family size	[16,21,24,30,32,33,36,37,38,39,41,45,46,47]
	Nuclear family	[44]
	Depend on agricultural land	[16]
	Irregular work of father	[56]
	Unemployed father	[46]
	Maternal employment	[39]
	Those who have lost their mother	[46]
	The age at which the child starts complementary foods	[16]
	Children whose parents were laborers	[20]
	Being younger	[17,41,47]
	Older age group	[55,56]
	Adolescent who come from male-headed household	[17]
	Low educational status of parents	[17,21,23,26,29,32,33,34,36,37,43,44,45,46,48,49,50,51,52,53]
	Formal education	[54]
	Type of school attended	[42]
	Being female	[39,48]
	Being male	[38,47]
Malaise	Those who suffered diarrhea	[68]
	Those who suffered cold/coughs	[68]
	Anorexia	[52]
Nature of environment	Children who live in flood areas	[18]
	Poor environment	[26]
	Living slum	[36,57]
	Rural environment	[28]
	Urban environment	[28,44,58]
	Living in small houses	[39]
Sanitation	Unsafe drinking water	[16,54]
	Children with poor personal hygiene	[59]
Knowledge of practice	Source of information	[60]
	Low information on nutritional panel	[20,23,24,60,61]
	Poor breastfeeding practices	[16]
Dietary Habit	Adolescent who consume regular carbonated soft drinks	[13,62]
	Poor eating habits	[36]
	Adolescents who consume regular fast food	[13,62]
	Households without food security	[13,37,48,50,62]
	Inadequate dietary intake of adolescent	[17,27,31,42,47,52,63,65,67,68]
	Adolescents who consumed diet of low diversity	[17,25,68]
	Adolescents who consume foods such as plus, legumes, and lentils	[71]
	Children with low consumption of high-quality protein	[20,30,41,56,63,64,65,66]
	Poor dietary quality	[67]
	Children who have the habit of eating in between meals	[69]
	Children who are having dinner as the heaviest meal of the day	[69]
	Children who consume more than three meals in a day	[69]
	Micronutrient deficiency	[73]
	Skipping breakfast	[30,56,70]
	Children who consumed beverages between meals per day	[54]
	Early cessation of breast feeding	[52]
Nutrient Deficiency	Vitamin A	[41,65,66,72]
	Calcium deficiency	[65,66]
	Zinc deficiency	[66,72]
	Iodine deficiency	[72]
	Iron deficiency	[66,72]
	Micro-nutrient deficiency	[52]
	Maternal malnutrition	[52]
Body Weight	Obesity	[74]
	Overweight	[74]
Means of transport	Children who use vehicular transport to school	[69]
Household activities	Depending on family members	[60]
	Not participating in household activities	[69]
	Children who watch TV more than 3 hours per day	[69]
Sedentary lifestyle	Children who have the habit of not playing outdoor games	[69]
	Adolescents who do not do any physical activities	[69]

**Table 4:** Showing health impact of malnutrition among in-school children in developing countries.

Main Theme	Subtheme	Authors
General health status	Malaria	[20]
	Headaches	[20]
	Nose bleeding	[20]
	Abdominal pains	[20,76]
	Fainting	[20]
	Pallor	[59]
	Myopia	[59]
	Diarrhea	[68]
	Colds/coughs	[68]
	Vomiting	[68]
	Fever	[68]
Fetal/Maternal health	Poor maternal health	[13]
	Preterm birth	[13]
	Risk of Small for Gestational Age (SGA)	[13]
Morbidity	Increased morbidity in children	[67]
Social issue	Gender discrimination	[16]
Eye (Ophthalmologic) Complications	Conjunctival xerosis	[35]
	Bitot’s spot	[35]
	Dental caries	[59,63,76]
	Physiological and developmental delays	[28]
Cognitive function	Reduced cognitive functions	[28,50,52,64,70,72,74]
Metabolic Risk factors	Obesity	[21,64,74]
	Overweight	[21,74]
	Risk of nutrition-related chronic diseases	[33]
Immunity/Infection	Lowered resistance to infection	[52,61]
	Recurring illness	[13]
	Upper Respiratory Infections (URI)	[20,76]
	Lower Respiratory Tract infection	[76]
	Skin infection	[20,68]
	Risk of developing non-communicable diseases	[21,69]
	Parasitic infections	[40,76]
	Impaired immune function	[28]
Nutritional deficiency	Vitamin A deficiency	[18,19,22,35,40]
	Vitamin B complex deficiency	[18]
	Iron deficiency	[18,22,40,66,73]
	Calcium deficiency	[63]
	Inadequate dietary intake	[40]
Growth Retardation	Faltering growth	[61,64]
	Long-term relative physical growth retardation	[28]

**Table 5:** Showing impact of malnutrition on school performance and attendance of in-school children in developing countries.

Main Theme	Subtheme	Authors
School Performance	Diminished learning ability	[18,61]
	Work efficiency	[18]
	Poor academic performance	[27,32,42,52,53,67,70,77,78,79]
	Lack of concentration	[32]
	Poor memory	[52]
Attendance	High levels of school dropout	[20,32,42,74]
	Low-class attendance	[32,42,64,72,74,80]
	Delayed school entry	[27,32,42]
	Decreased graduation rates from primary and secondary school	[27]

## Data Availability

Not applicable.

## Supplementary Materials

The following supporting information can be downloaded at: https://www.mdpi.com/article/10.3390/children11040476/s1, Table S1: Data extracted from studies included. References [93,94] cited in Supplementary Materials.
