# Supplementary material for: Risk Factors of Malnutrition among In-School Children and Adolescents in Developing Countries: A Scoping Review
Source: Children (Basel). 2024 Apr 15;11(4):476. doi: 10.3390/children11040476 (PMC11049343; doi:10.3390/children11040476)
Supplement: Supplementary file 1 [file children-11-00476-s001.zip › children-2933232-supplementary.pdf]

Table S1. Data extracted from studies included.

Authors, Year and Country	Purpose of the Study	Study Design	Population	Sample Size	Risk Factors	Health Impact	Impact on School Performance and Attendance
[29] India	To estimate the prevalence of malnutrition among school-going adolescent children. To assess the correlation between nutritional status of adolescent children and their parents' literacy level and socio-economic class.	Cross-sectional	Among school-going adolescent children	1000	Children whose mothers and fathers were illiterate. Low socio-economic status of the parents.		
[13] Bangladesh	This study focused on investigating the extent of malnutrition, including individual-level double burden and associated factors influencing malnutrition amid school-going adolescents of Bangladesh using 2014 Bangladesh GSHS survey data. And examined individual-level double burden (coexistence of stunting and overweight at	Cross-sectional survey	School-going adolescents	2989	Adolescents who consume regular carbonated soft drink, adolescents who consume regular fast food, households without food security.	Poverty, poor maternal health, recurring illness, risk of Small for Gestational Age (SGA) and preterm birth.	

	individual level).						
[16] Bangladesh	To assess the prevalence of malnutrition among primary school-going children and its associated factors.	Cross-sectional survey	Primary school children	400	Poor breast-feeding practices, low total family income, unsafe drinking water. The age at which the child starts complementary foods, Large family size, parents who depend on agricultural land.	Gender discrimination (where boys are given higher importance than girls) leads to the boys receiving more nutrition and proper care than girls.	
[17] Ethiopia	The aim of this study was to assess nutritional status and associated factors among school adolescents in Chiro town.	Cross-sectional study	School adolescent	291	Inadequate dietary intake of adolescent, adolescents who consumed diet of low diversity, being younger due inadequate income, adolescents who come from male-headed household, and low educational status affect income and knowledge about nutrition.		
[18] India	To assess the nutritional status of school-going children in a village.	Cross-sectional survey	School-going children	600	Children who come from weak socio-economic background, children who live in flood areas.	Vitamin A deficiency was noted as the cause of night blindness, conjunctival, xerosis, and corneal xerosis. Vitamin B-complex deficiency was noted as the cause of angular stomatitis, glossitis and iron	Poor learning ability and reduced work efficiency. It affects intellectual development. It increases the chances of children becoming poor reader and poor writers.

						deficiency were noted as the causes of anemia in children.	
[19] India	To find out the prevalence of vitamin A deficiency (VAD) among school-going children aged 6–12 years and to assess the nutritional status of these selected school-going children.		School-going children aged 6–12 years	30	Children belonging to lower socio-economic class are prone to malnutrition.	Exhibition of conjunctival xerosis, night blindness.	
[20] Kenya	To determine the level of nutrition knowledge, attitude and practices and nutritional status of school-going children in a semi-arid food-insecure region.	Cross-sectional study	School- going children	350	Children who drop out of school were at risk due to lacking nutritional education, children with low consumption of high-quality protein. Children of parents with low-ranking occupation status were at risk; causal labor livelihood among most households.	Poor sanitation leading to disease prevalence examples ; the most common diseases among school children were malaria, headaches, noose bleeding, fainting, stomachache and diseases of the Upper Respiratory Infections (URI). Others were skin infections, worm infestations, pneumonia and wounds that took long to heal.	high levels of school dropout and educational wastage,
[21] India	To assess the prevalence and certain socio-economic and demographic factors affecting the double burden of malnutrition	Cross-sectional study	Urban school-going children	1017	Low-income level of household, low standard of education of mothers, large family size.	Acceleration of preventable, non-communicable diseases and higher prevalence of overweight–obesity and slightly lower prevalence of under nutrition.	

	among urban children.						
[22] Burkina	To assess the nutritional status of schoolchildren attending public and private schools.	Cross-sectional study	School-going children	649	Children whose parents are poor or of lower-income families are at risk; iron deficiency in children.	Micronutrient malnutrition, namely anemia and vitamin A deficiency, were highly prevalent.	It was observed that several school-going children stayed at school during lunchtime, but did not have pocket money to buy any street food, or did not have enough to eat an adequate meal.
[23] Mauritius	To determine whether specific socio-economic factors were associated with poor nutritional status among children.	Cross-sectional survey	School-going children	240	Parents who had low level of education, including poor nutrition knowledge, leading children to be at risk.<br>Families who are poor cannot afford expensive food commodities to their children.		
[24] Kenya	To assess the nutritional status and associated factors among school-going children.	Descriptive cross-sectional study	Among school-going children	315	Children who live in large-size households.<br>Children who come from low-income households.<br>Poor knowledge of mothers about a child’s nutrition.		
[25] Nigeria	To determining if differences exist between them and providing a baseline data for the	Cross-sectional study	Young school children	415	Children in public schools were at risk due to the socio-economic background, types of food		

	state in this regard.				common in the environment and storage and preparation modalities, and the general environmental factors that pervade the state.		
[26] Nigeria	To compare the nutritional status of public primary school children in an upland and a riverine area of Ojo Local Government Area of Lagos State.	Cross-sectional study	School children	240	Children of others of low-income status and mothers who had little or no formal education were at risk. Children whose mothers have low socio-economic status and those living in poor environmental conditions were at risk.		
[27] Ethiopia	To identifying the level of malnutrition and associated factors among high school adolescents.	Cross-sectional study	School-going adolescent girls	726	Families with low income were at risk of stunting, families with better socio-economic status were also at risk of obesity, families whose children take inadequate energy-rich food were also at risk. Adolescents of fathers from lower levels of education had a significantly higher risk of being underweight.	It is known that short stature is associated with cephalopelvic disproportion and increased risk of maternal complications during child birth.	Stunting was associated with delayed school entry, repetition and dropout rates, as well as decreased graduation rates from primary and secondary school, and lower school performance.

[28] India	To evaluate the socio-economic and demographic determinants among school-going children belonging to the Rajbanshi population of North Bengal, India.	Cross-sectional study	Among school-going children	350	Children living in poverty, poor socio-economic status and rural environments were at risk of undernutrition, while those residing in urban environments and a higher socio-economic status were associated with overweight/obesity.	The prevalence of undernutrition is usually attributed to long-term relative physical growth retardation, physiological and developmental delays, impaired immune function and reduced cognitive functions.	
[30] Egypt	To assess the nutritional status, and identify the socio-demographic and lifestyle factors affecting it among rural school children.	Cross sectional survey	Among rural school children	736	Children with poor poultry consumption and those skipping breakfast were at risk; irregular work of the father, large family size, and low socio-economic standard were also some of the risk factors.		
[31] India	To assess the nutritional status of school-going tribal children using anthropometric indices.	Cross sectional survey	School-going tribal children	1221	Children who were from poor homes were at risk. Children who lack nutritious food were also at risk.		
[32] Egypt	To determine the nutritional status and dietary habits of school-aged children (6–12 years) attending public (non-fee	Cross-sectional descriptive	School-aged children	288	Children with large family size, low-income earners and low-level of education parents were at risk.		Health problems due to miserable nutritional status in school-aged children are among the most common causes of low school enrolment, high absenteeism, early

	paying—NFP) and private (fee paying—FP) primary schools.						dropout and unsatisfactory classroom performance
[33] 2016 India	To assess the nutritional status of 6–14-year-old school-going children and the association among socio-economic factors, living conditions and children's undernutrition.	Cross-sectional descriptive	School-going children	100	Low level of parent's education, large family size, and poor social economic status of parents kept children at risk.	Excessive weight gain later in childhood increases the risk of nutrition-related chronic diseases.	Poor health and nutritional status among school-going children may contribute to high rate of school dropout, absenteeism, and poor academic performance. Hungry school children tend to be nervous, irritable, disinterested and unable to fully concentrate in the classroom.
[34] Karnataka	To evaluate the level of malnutrition and the impact of households on the nutritional status of school children in a rural area.	Cross-sectional descriptive survey	School children	270	Poor socio-economic status of mothers put their children at risk, children whose mothers has lower or no education		
[35] India	To evaluate the prevalence of vitamin A deficiency among the primary school children in relation to socio-economic status.	Cross-sectional study	Primary school children	600	Lower socio-economic class were at risk	Vitamin A deficiency was observed in students, among them some had conjunctival xerosis and Bitot's spot.	
[36] Pakistan	To explore the nutritional status of school-going children.	Cross-sectional study	School-going children,	571	Children who live in urban slums areas were at risk.		

[37] Pakistan	To find the impact of socio-economic factors on nutritional status in primary school children.	Cross sectional and descriptive study	Primary school children	161	Children of family living in poverty, with low literacy rate, of large families, and experiencing food insecurity were at risk
[38] 2019 Nepal	To find out the prevalence and associated factors of malnutrition among school-going adolescents.	Descriptive cross-sectional	Among school-going adolescents	510	Adolescents living in joint families were at risk; family with no earning status were also at risk. Male adolescents were found to be more at risk of being overweight and obese then female adolescents.
[39] Sri Lanka	To determine socio-economic factors associated with nutritional status among children in plantation communities.	Cross-sectional	Among preschool and school-going children	547	Maternal employment, high number of siblings, high birth orders and female children were significantly associated with undernutrition among preschool children, while living in small houses, large number of family members, low monthly income, and maternal employment were significantly associated with undernutrition

					among school children.		
[40] Nigeria	To determine the prevalence of stunting, thinness; vitamin A and iron deficiencies among adolescent students and to determine factors that are associated with these nutritional problems.	Cross-sectional study	Among adolescent students	400	Households with low income were more at risk to be vitamin-A-deficient.	VAD has been associated with anemia. Inadequate dietary intake, parasitic infections and menstrual losses are major causes of iron deficiency anemia.	
[41] Nigerian	To determine the prevalence and predictors of under-nutrition among school children.	Cross-sectional	School children	450	Children with zinc deficiency and vitamin A deficiency were at risk; household income, weekly food expenditure, and age of the child were some of the other risk factors.		
[42] Ghana	To investigate the determinants of malnutrition among pupils attending public and private schools	Cross-sectional survey	Among pupils attending school	633	Family with lower socio-economic status were at risk; locations of pupils (rural versus urban), guardian's occupation, and number of meals consumed in a day and the type of school attended were the other risk factors.		Malnutrition is the cause of low school enrolment, high absenteeism, early dropout and unsatisfactory classroom performance in school children
[43] India	To assess the socio-economic	Cross-sectional	Among school children	465	Socio-demographic factors such as caste,		

	predisposing factors of malnutrition among school children.				and socio-economic factors such as parental education, employment and socio-economic conditions were the risk factors.	
[44] India	To determine the prevalence of underweight, overweight, and obesity among school-going adolescent girls along with dietary and physical activity-related factors associated with them.	Cross-sectional study	School-going adolescent girls	2400	Girls belonging to middle- and upper-socio-economic strata were more at risk of obesity. Adolescent females who belong to nuclear families were at risk of overweight. Adolescents who reside in urban areas were more at risk of overweight and obesity compared to rural one. Those with their father's education of middle school and above were more at risk of overweight and obese as compared to the adolescents with father's education at the maximum.	
[45] Egypt	To determine the nutritional status and dietary habits of	Cross sectional survey	School Children	1100	Low level of both parents' education and large family size were the risk factors.	

	school children.				
[46] India	To determine the burden and possible predictors of malnutrition among primary and upper-primary school-aged children.	Cross-sectional	Among primary and upper-primary school-aged children	24,108	Subjects with three or more siblings were at risk and unemployed father, illiterate mother or father and those who had lost their mothers were also at risk.<br>Children with leave in rural areas were also at risk.
[47] Ethiopia	To asses the prevalence of under-nutrition and its associated factors among children.	Cross-sectional	Among children	459	Children with large family size were at risk.<br>Children who had fewer frequent meals were also at risk, and those within the age of 10 to 14 years were also at risk; male children were more at risk than female children.
[48] India	To estimate the prevalence and risk factors of malnutrition among school-going children	Cross-sectional study	School-going children	150	Children from illiterate mothers were at risk,<br>children who were experiencing lack of healthy sustenance from a joint family were also at risk,<br>and female students were more at risk

					than male students.		
[49] India	The study was carried out to assess the nutritional status of school children between the ages of 6 of 12 years.	Cross-sectional study	School-going children	284	Children whose parents are illiterate stand the chance of being malnourished.		
[50] Nepal	To study the nutritional status assessment of adolescent school children.	Cross-sectional study	Adolescent school-going children	192	Families who have difficulty in accessing food. Low level of mother's education.		It can cause cognitive effects.
[51] India	To estimate the magnitude of malnutrition among school-going children in a slum area of Kolkata and to elicit the association of nutritional status with parental education.	Cross-sectional observational study	School-going children	508	Children with parents who have poor educational backgrounds.		
[52] India	To explore protein energy malnutrition related learning disabilities in school-going children in relation to the Prakrit.	Cross-sectional design	School-going children	1520	Children with inadequate dietary intake, anorexia, malabsorption, micro-nutrient deficiency, early cessation of breast feeding, maternal illiteracy, maternal malnutrition, and poverty were at risk of protein energy malnutrition.	Malnutrition was responsible for the susceptibility towards a variety of physical and mental diseases including the development of various infectious diseases.	Poor cognitive function, particularly memory and learning performance, was found to be associated with malnourished children.

[53] Pakistan	To assess the nutritional assessment of the primary school children in Abbottabad.	Descriptive cross-sectional study	Primary school children	200	Children with lower socio-economic status were at risk.	Children of well-serviced fathers and qualified mothers were healthy, more intelligent and practiced good hygiene.	Children attending a private school who have better nutritional status perform better than public school children.
[54] Ghana	To determine the prevalence of undernutrition and overweight/obesity and its associated factors among children.	Cross-sectional study	School children	423	Children whose household used water from non-portable sources were at risk of being undernourished. Children whose mothers had attained formal education were at risk of overweight/obesity. Children who consumed beverages between meals per day were also at risk of overweight/obesity.		
[55] Burkina Faso	To determine the prevalence of undernutrition and its risk factors among schoolchildren in Burkina Faso before the start of the project.	Cross-sectional	School children	455	Older-age group (12–14 years) were at risk compared to those younger than 11 years, and children with middle or lower hygiene categories were also at risk.	WASH, health education and nutritional interventions will be implemented with the goal of improving schoolchildren's health.	
[56] Egypt	To assess the level of malnutrition and associated factors among	Cross-sectional study	School children	736	Increasing age, reduced poultry consumption, and skipping breakfast	Prevalence of obesity was significantly higher in younger age group	

	school children.				were associated risk factors.	compare with older age.
[57] Bangladesh	To propose a new approach to solve nutritional deficiency of school-going children in Bangladesh.<br>◎ To determine the prevalence of underweight, stunting and wasting in school-going urban and slum children of Chittagong, Bangladesh.<br>◎ To analyze factors correlated with malnutrition in school-going children.	Cross-sectional study	School-going children	50	Children living in slum areas were at risk.	
[58] Pakistan	To determine the frequency of nutritional status and socio-demographic factors influencing under nutrition among school children of rural Islamabad.	Cross-sectional study	Among school children	1710	Children in urban areas are at risk of having greater weight and height than those in rural areas.	
[59] India	To assess the various morbidities and nutritional status among school children.	Cross-sectional study	Among school children	714	Children with poor personal hygiene.	Some of the common conditions suffered by these children include pallor, myopia, and dental caries.

[60] Bangladesh	To identify the sources of information and corresponding knowledge level on nutrition among school-going adolescents.	Cross-sectional survey	School-going adolescent	855	Those who source information online, those with low information on the nutrition facts' panel, and those depending on family members were at risk.		
[61] Western Nepal	To find the prevalence of malnutrition among school children 4-14 years old and the effect of socio-demographic characteristic of mothers on child nutrition.	Descriptive Cross-sectional survey	School children	789	Children whose mothers had a poor knowledge of diet, and children whose mothers were laborers were at risk.	Lowered resistance to infection (diarrheal diseases and respiratory infections),<br>faltering growth.	Diminished learning ability.
[62] Benin, Djibouti, Egypt, Ghana, Malawi, Mauritania, Morocco	To evaluate the prevalence of underweight, overweight, and obesity as well as associated risk factors among school-going adolescents.	Cross-sectional study	School-going adolescent	25815	Households with food insecurity, adolescents who consume fast food and soft drinks, adolescents who do not do any physical activities.		
[63] Pakistan	To assessment of nutritional status among school-going children through their dietary intake.	Cross-sectional study	Among school-going children	200	Inadequate consumption of food groups by children, inadequate milk intake.	Due to low consumption of dairy products, children are at risk of developing osteoporosis on later age. Calcium is required for bones growth and height.<br>Consumed candies/chocolates daily, which is the main reason for developing dental	

						carries and childhood obesity.	
[64] Kenya	To determine the household food and nutrition security among households with school-going children.	Cross-sectional Study	School-going children.	386	Children who do not consume livestock products are at risk.	It affects their physical and cognitive development.	It affects their physical and cognitive development leading to low-class attendance hence poor school performance
[65] 2019 Botswana	To determine the effect of the Botswana School Feeding Programme (SFP) (all government schools) on the nutritional status of primary school children in the South-east District when compared to non-beneficiaries of the SFP (private schools).	Comparative cross-sectional study	Among primary school children	392	Children who have inadequate supply of energy, protein, vitamin A and calcium were at risk.		
[66] Ghana	To determine the Nutrition intakes and nutritional status of school-aged children in Ghana school-aged children	Comparative cross sectional	School-aged children	425	Children with low intake of protein, zinc, iron, calcium, vitamin A and vitamin C were at risk	At least 3 out of 10 school-age children were anaemic	
[67] Ghana	To assess the influence of Ghana School Feeding Programme on nutritional status of school children	Case control study	Participating and non-participating pupils in the Ghana School Feeding Programme	234	inadequate food intake of children, poor dietary quality and increased morbidity in children	It may result in morbidity and fewer opportunities for economic development, adversely affect their mental development	poor academic performance OR adversely affect their cognitive

[68] Kenya	To assess the nutrition status and associated risk factors of children in selected public primary schools	Cross-sectional design	Among school-aged children	208	Children who had diarrhoea, colds/coughs are at risk. Children who took inadequate energy food are risk. Those who took less than four varieties of food.	Incidence of diarrhoea, colds/coughs vomiting, fever and skin rashes. increased due to malnutrition	
[69] India	To study the diet and physical activity factors influencing overweight and obesity in school-aged children.	Cross-sectional study	School-aged children	400	Children who have the habit of eating in between meals, Children who are having dinner as the heaviest meal of the day, Children who consume more than three meals in a day, children who have the habit of not playing outdoor games, Not participating in household activities, Children who use vehicular transport to school, children who watch TV for more than 3 hours per day.	Risk of developing non-communicable diseases in adult life.	
[70] Iraq	To assess the nutritional status and nutritional habit of primary school children and to relate it with their Intelligence	Cross sectional survey	Primary school children	529	Children with bad food habits, and children skipping breakfast were also at risk.	Poor nutritional status can adversely affect brain function and impact cognition and behavior.	Malnutrition among children is still a public health issue in Iraq and it affects the children's cognitive function and academic performance at school.

	Quotient (IQ) score using standardized tools.						
[71] Ethiopia	To assess adolescent nutritional status and associated factors in secondary and preparatory school students.	Cross-sectional study	Secondary and preparatory school adolescent students	572	Adolescents who consume foods such as plus, legumes, and lentils were statistically at risks.		
[72] Senegal	To assess the anthropometric and micronutrient status of children from state schools in the Dakar area.	Cross sectional survey	School children	604	Children with deficiency vitamin A, zinc, iodine and iron were at risk of malnourishment.	Iodine, iron and folic acid micronutrient deficiencies affect the development of the brain and cognitive functions of school children.	Deficiencies of vitamin A and zinc are associated with different scenarios affecting school performance, such as absenteeism due to increased morbidity.
[73] Ghana	To assess and compare the nutritional status of boarding and non-boarding pupils aged 8 to 10 years.	Cross-sectional study	School-aged children	124	Children with micronutrients were found to be at risk.	About 72% of the children were found to be anemic due to iron deficiency.	
[74] Nigeria	To assess the physical growth and nutritional status of school children.	Cross-sectional study	School-going children	2616	Children who are obese and overweight are at risk.	Overweight and obesity have also been linked to increased risk of cardiovascular and pulmonary diseases.	Chronic undernutrition in older children is linked to slower cognitive development, poor school attendance, high school withdrawal rate.
[75] India	To evaluate the extent of underweight and stunting present among 1–12 year-old children belonging to a low socio-economic status.	Cross-sectional study	Pre-adolescent children of low socio-economic class	1206	Children with family with low income.		

[76] India	To identifying the commonly prevailing health and nutritional disorders in school-going children is essential to initiate appropriate public health measures.	Cross-sectional prospective study	School-going children	28,256	Worm infestation was the commonest morbidity found in most children, followed closely by upper respiratory tract infection and dental caries. Other diseases encountered were skin disorders, ear discharge, lower respiratory tract infections and abdominal pain.	
[77] Pakistan	To assess the prevalence nutritional status and correlation between nutritional status of students and the academic performance of school-going children.	Cross-sectional study	School-going children	400		Low level of educational performance in children who are malnourished.
[78] India	To determine the relationship between nutritional status and academic achievement of school children.	Cross-sectional study	School children	135		There was a positive and highly significant difference found between nutritional status with academic achievement; hence, increasing the nutritional status of children in turn increases their academic achievement.
[79] 2017 India	To assess the association of malnutrition with scholastic performance among 8–12-year children in two selected	Cross sectional study	School-going children	384		It was found that poor scholastic performance was significantly associated with underweight, stunting and BMI.

	private schools in Meerut. To assess the association of malnutrition with scholastic performance among selected school-going children.						
[80] Madagascar	To estimate the possible causalities between children's nutritional status and academic performances in rural Madagascar.	Cross-sectional	Among primary schoolchildren	399			School attendance rates among children not stunted and among those not underweight were significantly higher than among those stunted and among those underweight, respectively. No significant difference was identified in attendance rates between those who were thin and not thin and between those who were overweight and not overweight.
[81] Madagascar	To estimate prevalence of malnutrition among schoolchildren aged 5-14 years.	Cross-sectional	School children	393	Children between the ages of 5 and 14years were at risk, children in large household were also at risk, and children who do not consume adequate animal protein were also at risk.		

[82] Nigeria	To assess the iron, zinc and copper status of primary school-attending children.	Cross sectional survey	Primary school-attending children	200	
[83] India	To assess nutritional status of weight for age, height for age and weight for height based on anthropometric measurements.	Cross-sectional study	Among school-going boys and girls	446	
[84] Nepal	To assess the nutritional status in terms of prevalence of underweight, stunting and thinness among rural school-going children.	Cross-sectional study	Rural school-going children	438	
[87] Ethiopia	assessed the magnitude of adolescents under nutrition and its associated factors among primary and secondary public school	Cross-sectional	Adolescents	411	
[88] Nigeria	The objective of this research was to carry out the anthropometric measurements of the pupils; predict the nutritional status of the school children; carry out the	Experimental study	Pupils in a public primary school	160	Children who have low intake of vitamins from school meals were at risk.

	nutrient analysis of the school meals; and calculate the nutrient intake therefrom and then compare it with Recommended Dietary Allowance (RDA) for children.				
[89] India	To assess the prevalence of malnutrition among under-five children of the Kadukuruba tribe and socio-demographic factors associated with it.	Cross-sectional study	Under-five children	101	
[91] India	To determine the nutritional status by using four commonly used anthropometric indicators.	Cross-sectional	Among school children	200	
[92] Ghana	To compare the nutritional status of SAC enrolled in schools with the SFP and SAC enrolled in schools without the SFP in place for the purpose of identifying which group has the higher rate of malnutrition	Cross-sectional	School children	359	Sex of pupil, age of pupil, area of residence and community type were risk factors.

[93] India	To assess the overall prevalence of under nutrition using composite index of anthropometric failure.	Cross sectional study	Among school-going children	360	
[94] Pakistan	To understand the different factors which affect malnutrition on school-going children in rural communities.	Cross-sectional	School-going children	85	Children with low family incomes, low level of family education and poor eating habits were at risk.

Some cells were left blank because the studies included did not have information to fill such cells.
